# Photoelastic analysis of conventional and locking system for treatment of mandibular angle fractures with a single plate

**DOI:** 10.4317/jced.56916

**Published:** 2021-04-01

**Authors:** Danilo-Louzada de Oliveira, Victor-Eduardo de Souza-Batista, Letícia Holobenko, Joel-Ferreira Santiago-Junior, Eduardo-Piza Pellizzer, Paulo-Domingos Ribeiro-Junior

**Affiliations:** 1 Department of Oral and Maxillofacial Surgery, Universidade do Oeste Paulista (UNOESTE), R. José Bongiovani, 700, 19050-920, Presidente Prudente, SP, Brazil; 2 Department of Prosthodontics, UNOESTE, R. José Bongiovani, 700, 19050-920, Presidente Prudente, SP, Brazil; 3 Department of Health Sciences, Universidade do Sagrado Coração, R. Irmã Arminda, 10-50, 17011-160, Bauru, SP, Brazil; 4 Department of Dental Materials and Prosthodontics, School of Dentistry, Universidade Estadual Paulista (UNESP), Araçatuba, SP, Brazil. R. José Bonifacio, 793, 10-50, 16015-050, Araçatuba, SP, Brazil; 5 Department of Oral and Maxillofacial Surgery, Universidade do Sagrado Coração, R. Irmã Arminda, 10-50, 17011-160, Bauru, SP, Brazil

## Abstract

**Background:**

This photoelastic analysis evaluated stress distribution in different osteosynthesis systems, conventional and locking, used for treatment of mandibular angle fractures with a single plate.

**Material and Methods:**

Angle fractures were simulated in mandibles made of photoelastic resin. Following Champy’s method, plate osteosynthesis was performed. The samples were divided into five groups: Group 1, non-fractured mandible; Group 2, two screws were installed in each segment using a conventional system; Group 3, two screws were installed in each segment using a locking system; Group 4, three screws were installed in the proximal segment and four screws in the distal segment using a conventional plate; Group 5, three screws were installed in the proximal segment and four screws in the distal segment using a locking plate. In an universal testing machine coupled to a polariscope, a load was applied to the first molar 10 times. The 50 images were randomly numbered and analyzed qualitatively and quantitatively by two raters.

**Results:**

The locking system promoted better stress distribution along the osteosynthesis. The locking system reduced stress magnitude in the distal segment, with a significant between-group difference (*P*≤ 0.001).

**Conclusions:**

The locking plate/screw system can distribute stress more evenly throughout the osteosynthesis, especially when long seven-hole plates are used.

** Key words:**Internal fracture fixation, osteosynthesis, mandibular fracture, bone plates.

## Introduction

Mandibular angle fractures (MAF) have a specific pathophysiology that makes treatment challenging and prone to complications. Factors to be considered when treating these fractures include the particular anatomy and physiology of the mandibular angle where the major masticatory muscles are inserted, high masticatory load, thin bone with little medullary bone, and presence of third molars (1-3).

The main treatment is internal rigid fixation (IRF) with plates and screws. One mode of treatment involves the use of two bone plates, one at the superior border of the mandible (tension band), usually secured with monocortical screws, and one at the inferior border (base) secured with bicortical screws1. Another mode of treatment consists of the technique introduced by Champy *et al.* (4), involving the use of a single miniplate in the oblique line region secured with monocortical screws. Although the methods of fixation in the treatment of MAF remain debaTable (1,5-7), the use of a single miniplate appears to be a predicTable method (8-11).

The type of osteosynthesis system used is an important factor that may affect the outcome of MAF treatment (12-14). It has been suggested that the locking plate/screw system has advantages over the conventional plate/screw system by allowing the stress created by mastication to be absorbed by the plate with less pressure on the bone (15-17). Locking systems have been associated with greater resistance to MAF displacement in biomechanical testing (7).

In vitro and clinical studies have found that long locking miniplates provide greater resistance when Champy’s method is used for MAF treatment (7,11). However, it remains unclear how stress is distributed in the region among the different types of osteosynthesis. The purpose of this study was to evaluate stress distribution in different types of straight plate osteosynthesis used for MAF treatment by means of photoelastic analysis.

## Material and Methods

A replica of a human dentate mandible made of rigid polyurethane (Nacional Ossos; Franceschi & Costa e Silva Ltda., Jaú, SP, Brazil) was used as a study model. The mandible was sectioned in the sagittal midline, and the right hemimandible was molded with a silicone-based material (Silibor®; Clássico Artigos Odontológicos Ltda., São Paulo, SP, Brazil) according to the manufacturer’s instructions.

This mold was used to fabricate five photoelastic analogs of the hemimandible using photoelastic resin (Araldite; Araltec Produtos Químicos Ltda., Guarulhos, SP, Brazil). The photoelastic models were constructed by mixing 100 parts of GY-279 and 48 parts of HY 2963, for a total of 40 mL used for each hemimandible in this experiment. Using a syringe, the manipulated resin was slowly injected into the mold through posterior openings corresponding to the posterior portion of the condyle until filling up the mold. After an additional 72 hours, the five photoelastic models were obtained. The hemimandibles were sectioned at the mandibular angle, simulating a linear fracture. The sections (fractures) were standardized by using an acrylic jig as described by Yamaji *et al.* (18).

**Figure 1:**
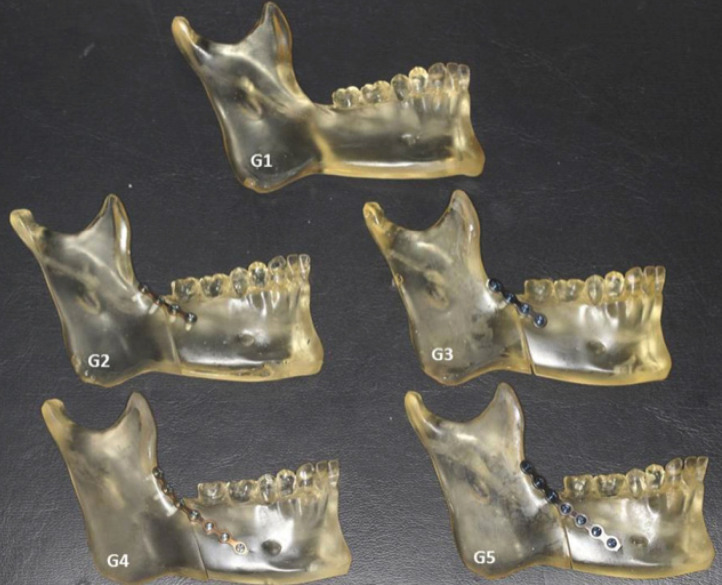
Photoelastic models. Non-fractured control hemimandible (G1) and fractured hemimandibles fixed by different osteosynthesis methods (G2: four-screw conventional plate, G3: four-screw locking plate, G4: seven-screw conventional plate, and G5: seven-screw locking plate).

The mandibles were divided into five groups. In Group 1 (G1), the mandible was left intact and used as a control in the photoelastic test (19). In the other four groups, a single straight 1-mm-thick plate of the 2.0 mm system (Sistema Neoface, Neoortho Produtos Ortopédicos S.A., Curitiba, PR, Brazil) was adapted to the oblique line of the fractured mandible, and the bone segments were fixed with 2.0 mm diameter × 6 mm long monocortical screws: Group 2 (G2), two screws were installed in each segment using a conventional system; Group 3 (G3), two screws were installed in each segment using a locking system; Group 4 (G4), three screws were installed in the proximal segment and four screws were installed in the distal segment using a seven-hole conventional plate; and Group 5 (G5), three screws were installed in the proximal segment and four screws were installed in the distal segment using a seven-hole locking plate (Fig. 1).

Photoelastic testing was performed in an Instron model 4411 universal testing machine (Instron Corp, Norwood, MA, USA) coupled to a circular polariscope. The hemimandibles were placed on a metal platform, and the condyle and the posterior border of the ascending ramus of the mandible were secured so that the occlusal plane formed an angle of 90° with the load cell.

Before load application, all models were checked for absence of stress. An increasing load up to 100 N was applied to the occlusal plane of the mandibular first molar, simulating masticatory forces, in order to analyze the distribution of stress fringes in the model (7,20,21). Whenever the final load was achieved, the displacement was determined and a photograph was taken for assessment of the distribution of isochromatic fringes. Each loading sequence was repeated 10 times in each hemimandible, for a total of 50 load applications in this study (22).

Images were analyzed by two raters, and inter-rater reliability was determined using Kappa statistic (k ≥ 0.8 indicates almost perfect agreement). Intra-rater reliability was determined by later reassessment of 20% of the images, according to a previously published protocol (22).

The 50 images were numbered in a random order using Research Randomizer 4.023 and randomly analyzed qualitatively and quantitatively on the computer screen by two independent calibrated raters blind to experimental conditions.

-Qualitative analysis

A qualitative approach was used for a descriptive and comparative analysis of the location, distribution, and concentration of fringes formed after each loading sequence. Fringe intensity was measured in the proximal and distal segments and in the MAF region. The magnitude of the fringes was measured as described in the literature (22), and the fringes were classified according to their intensity as low intensity (n = 1, red/blue transition), moderate intensity (n = 2, red/green transition), and high intensity (n = 3, pink/green transition)22.

-Quantitative analysis

Fringe intensity was analyzed quantitatively by summing the number of fringes.

The coefficient of fringes was used according to previous study (22) to determine the magnitude of stress, in which, after the identification and counting of the fringe order, a weight was attributed for each order. This weight was used to individualize the influence of each fringe order (stress magnitude) in the stress distribution, without masking the results. A fringe coefficient was calculated to show the higher intensity fringes by applying a multiplication factor to low-intensity (×1), moderate-intensity (×2), and high-intensity (×3) fringes. In this analysis, the number of fringes and the fringe coefficient in the proximal and distal segments and in the MAF region were scored as low, medium, or high intensity (22).

-Statistical analysis

The data resulting from load application and the analysis of fringes formed after each loading sequence were entered into an Excel spreadsheet (Microsoft Office Excel, Redmond, WA, USA) and then exported to SigmaPlot version 12.0 (SigmaPlot, San Jose, CA, USA) for statistical analysis. The normality of data distribution was applied, and the analysis of variance and Tukey’s post hoc test was applied to assess the differences in the number of fringes. The intra- correlation coefficient (ICC) was calculated to examine the degree of the intra-examiner. The level of agreement before and after the evaluators, analyzing 20 % of the whole sample again. It was calculated the systematic error (paired t test *p* > 0.05) and random error analysis (Dahlberg error). The correlation between total number of fringes and fringe coefficient was assessed by Spearman correlation and linear regression. The level of significance was set at 5% for all analyses.

## Results

Inter-rater agreement was almost perfect (k = 0.94). The before-and-after (intra-rater) analysis showed no significant difference in the number of fringes (*P* = 0.673; Dahlberg error = 1.13) or in the fringe coefficient (*P* = 0.836; Dahlberg error = 2.30).

**Figure 2:**
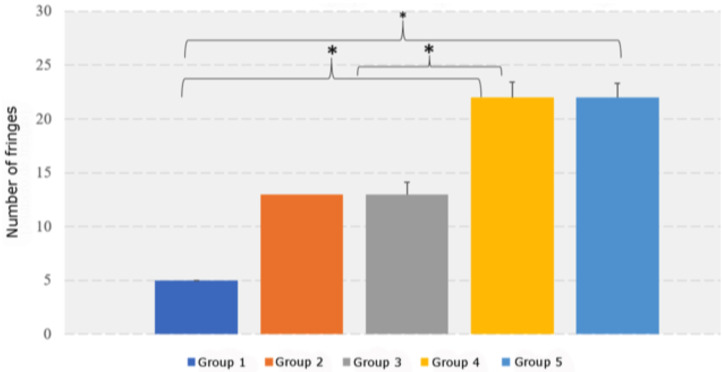
Between-group differences in number of fringes. * *P* < 0.05.

Overall, there were significant differences between the groups (*P* ≤ 0.001). Tukey’s post-hoc test showed significant between-group differences in the number of fringes for the following comparisons: G4 (mean, 22) vs G1 (mean, 5); G4 vs G3 (mean, 13); and G5 (mean, 22) vs G1 (Table 1, Fig. 2).

**Table 1:**
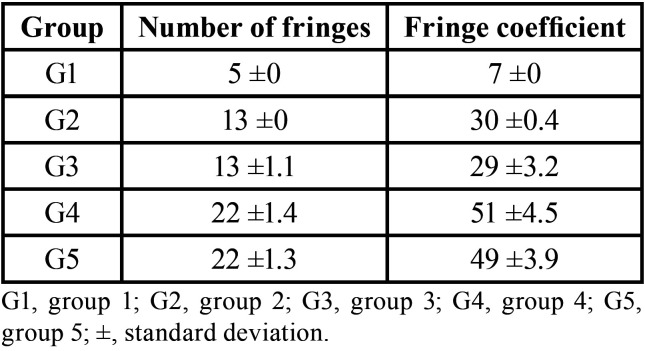
Mean and standard deviation of number of fringes and fringe coefficient.

**Figure 3:**
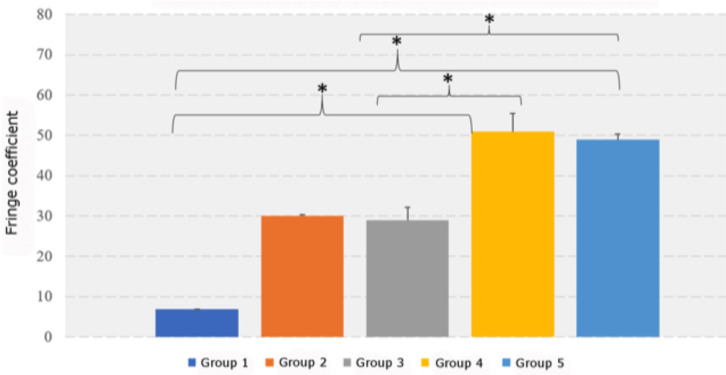
Between-group differences in the fringe coefficient. * *P* < 0.05.

There were also significant differences between the groups in the fringe coefficient (P ≤ 0.001). Tukey’s post-hoc test showed significant between-group differences for the following comparisons: G4 (mean, 51) vs G1 (mean, 7); G4 vs G3 (mean, 29); G5 (mean, 49) vs G1; and G5 vs G3 (Table 1, Fig. 3).

The distribution pattern of the number of fringes did not differ significantly between G2 and G3 or between G4 and G5. The same lack of between-group difference is true for fringe coefficient.

A correlation analysis between the photoelastic models found a very strong correlation between the number of fringes and fringe coefficient (r = 0.98), resulting in the following linear regression formula: Coefficient = −4.118 + (2.492 × No. Fringes).

In a specific analysis of the fringe coefficient per region of interest (proximal segment, distal segment, and fracture region), G1 (control group) showed significant differences between regions (*P*  ≤ 0.001). Tukey’s post-hoc test showed significant differences in the following comparisons: proximal segment (mean, 1) vs fracture region (mean, 3) and distal segment (mean, 3) vs proximal segment (*P*  < 0.05).

G2 showed significant differences in the fringe coefficient between regions (*P*  ≤ 0.001), with a significant difference in the following comparisons: distal segment (mean, 15) vs proximal segment (mean, 6) and distal segment vs fracture region (mean, 9) (*P* ≤ 0.001; Tukey’s post-hoc test). G3 also showed significant differences between regions (*P* < 0.05), with a significant difference in the comparison between fracture region (median, 11) and proximal segment (mean, 9) (*P* < 0.05; Tukey’s post-hoc test). When comparing G2 vs G3, G3 showed an increased fringe coefficient in the proximal segment and a decreased fringe coefficient in the distal segment (mean, 9).

G4 showed significant differences in the fringe coefficient between regions (*P* = 0.002), with a significant difference in the following comparisons: proximal segment (mean, 21) vs fracture region (mean, 12) and distal segment (mean, 18) vs fracture region (*P* < 0.05; Tukey’s post-hoc test). G5 also showed significant differences between regions (*P* ≤ 0.001), with a significant difference in the following comparisons: proximal segment (mean, 20) vs fracture region (mean, 9) and distal segment (mean, 21) vs fracture region (*P* < 0.05; Tukey’s post-hoc test). When comparing G4 and G5, G5 showed a decreased fringe coefficient in the proximal segment and fracture region as well as an increased fringe coefficient in the distal segment.

## Discussion

In the present study, the four types of osteosynthesis systems evaluated showed different patterns of stress distribution. This result was expected because the locking systems and long plates in this region have previously shown benefits (7,11). However, despite the benefits described in previous studies with the use of the locking system over the conventional system, it was unknown, until the present study, how bone stress distribution occurred in different osteosynthesis systems in models simulating MAF. It was also not known whether there was a difference in the distribution of stress in the different sites of osteosynthesis. This study was able to demonstrate that there is a difference in stress distribution among the different sites of osteosynthesis in a model simulating MAF.

Photoelastic testing allows the qualitative analysis of stress within a model by observing isochromatic fringes formed in the model (24), being particularly suitable for the analysis of deformations of complex structures such as the mandible (25).

Studies using photoelastic analogs of the mandible produce load-displacement curves similar to those observed in cadaveric mandibles, indicating that, under loading, the mechanical behavior of the analog is similar to that of the human mandible (26). These data favor the model used in the present study, as it is representative of the clinical situation. The biomechanical behavior of osteosynthesis devices tested in photoelastic models is similar to the performance of these same devices when used in facial fractures (25).

Studies have shown good results using the locking system, with an advantage in resistance over the conventional system (7,15-17,27-29). In the present study, the short plates by conventional (G2) and locking (G3) systems did not differ from each other in the distribution of stress fringes, and neither did long plates by conventional (G4) and locking (G5) systems. Likewise, when analyzing the fringe coefficient, no significant difference was observed in median values between G2 and G3 or between G4 and G5.

However, when the fringe coefficients in the proximal segment, distal segment, and fracture region were compared between G2 and G3, in G3 there was an increase in the proximal segment and a decrease in the distal segment. The same comparison between G4 and G5 showed a reduction in the number of fringes in the proximal segment and fracture region, but fewer fringes were found in the fracture region for G5 than for G4. This result suggests that long plates, with seven screws, by conventional systems (G4) may be more likely to be subjected to bone stress, increasing the chance of screw loosening. The long plates with locking system (G5) showed lower bone stress in the fracture region, thereby promoting load distribution across the region. Nevertheless, these data resulted from a qualitative analysis. When stress magnitudes were compared to each other, in a quantitative manner, no significant differences were found between locking and conventional groups.

In comparisons of the type of osteosynthesis systems used for MAF treatment, locking plate/screw systems have been shown to provide greater resistance to displacement, while long locking plates improve stability, although not significantly (7). Clinical studies of the treatment of MAF by Champy’s technique have shown good results with the use of long plates (11). In the present study, long locking plates provided better load distribution in all areas of the fracture, leading us to speculate that their use would promote an earlier fracture healing.

In all systems evaluated in the present study, an increase in stress fringes was observed in the screws placed close to the fractures, showing that bone stress is located closer to the fracture, increasing the likelihood of screw loosening and subsequent failure of the fixation system. Therefore, using screws of the locking system closer to the fracture may be a clinically preferable option, and also because the use of monocortical screws is recommended in this region. These findings are consistent with the report by Yi *et al.* (30) that, in photoelastic models simulating mandibular fractures and using reconstruction plates and bicortical screws, the screws placed closest to and farthest from the mandibular fracture would always bear most force, transmitting this force to the surrounding bone, which clinically may be beneficial in promoting bone resorption.

In the models tested in the present study, it seems clear that stress occurs throughout the osteosynthesis system. When four-hole plates were fixed with conventional screws (G2), stress was greater in the distal segment of the fracture, but when fixed with locking screws (G3), stress was reduced in the distal segment of the fracture, that is, a better load distribution was achieved along the osteosynthesis. As for seven-hole plates fixed with conventional screws (G4), stress was greater in the proximal segment, but when fixed with locking screws (G5), stress distribution was found to be similar in the proximal segment, distal segment, and fracture region. Therefore, the locking system was able to distribute stress more evenly throughout the osteosynthesis in all cases, which can improve stability as previously demonstrated biomechanically (7).

The use of long plates (seven-holes) in the osteosynthesis systems tested here promoted favorable stress dissipation compared with the use of short plates (four-holes). Therefore, it seems reasonable that surgeons would choose a locking and long plate/screw system that allows for the use of more than two screws in each bone segment in the presence of unfavorable local and systemic clinical findings in conjunction with MAF. In conclusion, the behavior of all models tested here suggests a possible clinical use. However, the locking plate/screw system seems most suiTable, especially when long miniplates are used.
